# Venous Thromboembolism Risk With Antidepressants: Driven by Disease or Drugs?

**DOI:** 10.1161/JAHA.117.006293

**Published:** 2017-05-17

**Authors:** Brian R. Branchford

**Affiliations:** ^1^ Section of Hematology/Oncology/Bone Marrow Transplant Department of Pediatrics University of Colorado School of Medicine Aurora CO; ^2^ Center for Cancer and Blood Disorders Children's Hospital Colorado Aurora CO; ^3^ University of Colorado Hemophilia and Thrombosis Center Aurora CO

**Keywords:** Editorials, antidepressants, depression, epidemiology, platelet, psychotropic drugs, risk factor, venous thromboembolism, Thrombosis, Epidemiology, Mental Health, Clinical Studies

## Introduction

The impetus for the article by Parkin et al in this issue of *JAHA* seems to stem from the authors' observation of the relatively common occurrence in women from the United Kingdom of (1) venous thromboembolism (VTE; deep vein thrombosis and/or pulmonary embolism, incidence of 0.8 per 1000 population per year), (2) depression (with or without the use of antidepressant drugs, 3.7% point prevalence), and (3) antidepressant drug use (to treat depression or other neurologic/gastrointestinal maladies, 11% usage rate), and from a desire to evaluate any associations among these conditions. The authors point out that some, but not all, previous studies in this area have suggested increased VTE risk with depression and/or antidepressant drug use, preventing true consensus on the matter. In their article, “Antidepressants, Depression, and Venous Thromboembolism Risk: Large Prospective Study of UK Women,” Parkin and colleagues attempted to overcome this ambiguity by taking advantage of the statistical power available from the large cohort size of the Million Women Study.^1^

Four main clinical groups were studied in this paper: women not taking antidepressants or being treated any other way for depression (*no treatment/no drugs*), those being treated for depression but not with antidepressants or other psychotropic drugs (*treatment/no drugs*), those being treated with antidepressant drugs (*antidepressants*), and those being treated with other psychotropic drugs (*other psychotropics*). Although these multiple groups may seem unwieldy at first, they provide a reasonable way to isolate effects of the individual factors separate from other variables. This arrangement of exposure categories, however, does not account for depressed women undergoing treatment (potentially underestimating the effect of depression itself) and does not differentiate between women using antidepressants for depression or for a neurologic/gastrointestinal disease.

To explore the relationships among antidepressants, depression, and VTE, the authors linked questionnaire data about depression and regular antidepressant use in 734 092 women with hospital admissions and deaths attributed to VTE who also fulfilled all inclusion criteria. Using this approach, they determined that antidepressant use is common in UK women (6.9% of the cohort, 59.4% of whom were being treated for depression or anxiety) and is associated with increased VTE risk (adjusted hazard ratio [HR]: 1.39; 95% confidence interval [CI], 1.23–1.56; *P*<0.0001) compared with women in the no treatment/no drugs group. Similarly, women in the other psychotropics group experienced significantly increased VTE risk (HR: 1.41; 95% CI, 1.19–1.67; *P*<0.0001) compared with the no treatment/no drugs group, but women in the treatment/no drugs group did not (HR: 1.19; 95% CI, 0.95–1.49; *P*=0.13). No difference was seen when restricting the analysis to women who had never smoked cigarettes. At first glance, these data seem to suggest that the antidepressant/psychotropic drug use drives the increased VTE risk rather than the depression itself.

Interestingly, the significantly increased VTE risk was observed for antidepressant users regardless of whether they also used other psychotropic drugs. In addition, the type of antidepressant did not seem to make a difference because the increased VTE risk was of similar magnitude for users of tricyclic antidepressants (adjusted HR: 1.32), selective serotonin reuptake inhibitors (HR: 1.40), and other antidepressants (HR: 1.61), even though these drugs are pharmacologically distinct. These facts could suggest that increased VTE risk may actually be related to the depression itself and not the treatment. This concept is supported by previous reports of depressive symptoms as a risk factor for recurrent VTE,^2^ likely through a prothrombotic platelet phenotype,^3^ possibly mediated by platelet‐derived microparticles.^4^

Risk‐factor associations like the ones discussed in the present study are important to ascertain, but the authors wisely stopped short of suggesting a causative relationship. It difficult to determine whether depression or antidepressant use drove the increased VTE risk or even if another related factor (eg, thrombophilia traits, use of other recreational or illicit drugs, presence of BDNF Val66met polymorphism leading to combined prothrombotic and depressive traits^5^) was the major impetus and caused some women to develop VTE, especially because—as the authors point out—the increased VTE risk was seen across several classes of antidepressants, including selective serotonin reuptake inhibitors, which have been reported to inhibit platelet function.^6^, ^7^, ^8^ This likely relates to the fact that serotonin is a weak platelet agonist but also potentiates stimulation from ADP or thrombin.^8^ When the reuptake of serotonin is blocked, it cannot be replaced within the dense granules of the platelet and thus is not available to augment the activation response^9^ or to help retain procoagulant proteins (fibrinogen, von Willebrand factor, thrombospondin, fibronectin, and α2‐antiplasmin) on their surface.^10^

Taken together, the data from this study suggest that women who reported taking antidepressants had a 40% higher risk of VTE development than women in the no treatment/no drugs group, regardless of which type of antidepressant or other psychotropic drug was used. The women in the treatment/no drugs group did not experience a significant increase in VTE risk. Consequently, the primary driving force of VTE development in this study seems to be the antidepressant or psychotropic drugs rather than the depression itself.

The authors identified and discussed 7 other studies with prospectively collected exposure information, 6 of which had similar findings, whereas 1 found no association but included a different type of control group for comparison. Similarly, 1 of 2 studies with retrospectively collected exposure data had results comparable to this study, whereas the other was again differentiated by control group. The authors' present study was unique in the evaluation of antidepressant use as a VTE risk factor because the other studies that were discussed either did not include this characteristic or classified the use in a way that might have resulted in underestimation of risk. In addition, the authors of the present study noted that their results of increased risk of VTE in users of other psychotropic drugs are consistent with findings from 6 other studies.

Because antidepressants are increasingly being used to treat depression, anxiety, and other gastric/neurologic conditions, it will be important for prescribers of these drugs to consider the increased VTE risk reported in this study. Similar to the screening questions about personal or family history of VTE recommended before estrogen‐containing hormone‐modulating therapy, providers may want to ask about VTE before prescribing antidepressants. Answers to these questions, and also ones hopefully being asked about bleeding history, given the previous studies on this topic,^7^ may help providers determine which class of antidepressant may be best for a particular patient (consistent with the general trend toward more personalized medication regimens).^8^ Nevertheless, more studies of the thrombotic and hemorrhagic potential of these drugs in various patient populations are needed before specific recommendations can be confidently expressed.

## Disclosures

None.

## References

[1] Parkin L , Balkwill A , Sweetland S , Reeves G , Green J , Beral V . Antidepressants, depression, and venous thrombembolism risk: large prospective study of UK women. J Am Heart Assoc. 2017;6:e005316 DOI: 10.1161/JAHA.116.005316. 28515116 10.1161/JAHA.116.005316 PMC5524086

[2] von Kanel R , Margani A , Stauber S , Meyer FA , Demarmels Biasiutti F , Vokt F , Wissmann T , Lammle B , Lukas PS . Depressive symptoms as a novel risk factor for recurrent venous thromboembolism: a longitudinal observational study in patients referred for thrombophilia investigation. PLoS One. 2015;10:e0125858. 25938663 10.1371/journal.pone.0125858 PMC4418654

[3] Lopez‐Vilchez I , Serra‐Millas M , Navarro V , Rosa Hernandez M , Villalta J , Diaz‐Ricart M , Gasto C , Escolar G , Galan AM . Prothrombotic platelet phenotype in major depression: downregulation by antidepressant treatment. J Affect Disord. 2014;159:39–45. 24679387 10.1016/j.jad.2014.02.022

[4] Williams MS , Rogers HL , Wang NY , Ziegelstein RC . Do platelet‐derived microparticles play a role in depression, inflammation, and acute coronary syndrome? Psychosomatics. 2014;55:252–260. 24374086 10.1016/j.psym.2013.09.004 PMC4004656

[5] Amadio P , Colombo GI , Tarantino E , Gianellini S , Ieraci A , Brioschi M , Banfi C , Werba JP , Parolari A , Lee FS , Tremoli E , Barbieri SS . BDNFVal66met polymorphism: a potential bridge between depression and thrombosis. Eur Heart J. 2015; 38:1426–1435. 10.1093/eurheartj/ehv655 PMC6251610 26705390

[6] Juurlink DN . Antidepressants, antiplatelets and bleeding: one more thing to worry about? CMAJ. 2011;183:1819–1820. 21969407 10.1503/cmaj.111576 PMC3216449

[7] Halperin D , Reber G . Influence of antidepressants on hemostasis. Dialogues Clin Neurosci. 2007;9:47–59. 17506225 10.31887/DCNS.2007.9.1/dhalperin PMC3181838

[8] Dietrich‐Muszalska A , Wachowicz B . Platelet haemostatic function in psychiatric disorders: effects of antidepressants and antipsychotic drugs. World J Biol Psychiatry. 2016;1–11. 10.3109/15622975.2016.1155748 27112326

[9] White JG . The dense bodies of human platelets. Origin of serotonin storage particles from platelet granules. Am J Pathol. 1968;53:791–808. 4176411 PMC2013516

[10] Dale GL , Friese P , Batar P , Hamilton SF , Reed GL , Jackson KW , Clemetson KJ , Alberio L . Stimulated platelets use serotonin to enhance their retention of procoagulant proteins on the cell surface. Nature. 2002;415:175–179. 11805836 10.1038/415175a

